# Effects of the Novel IDO Inhibitor DWG-1036 on the Behavior of Male and Female 3xTg-AD Mice

**DOI:** 10.3389/fphar.2019.01044

**Published:** 2019-09-24

**Authors:** Emre Fertan, Kurt R.J. Stover, Michael G. Brant, Paul M. Stafford, Brendan Kelly, Elena Diez-Cecilia, Aimée A. Wong, Donald F. Weaver, Richard E. Brown

**Affiliations:** ^1^Department of Psychology and Neuroscience, Dalhousie University, Halifax, NS, Canada; ^2^Krembil Research Institute, University Health Network, Toronto, ON, Canada

**Keywords:** Alzheimer’s disease, kynurenine pathway, quinolinic acid, behavior, mouse models, novel therapeutic

## Abstract

The kynurenine pathway metabolizes tryptophan into nicotinamide adenine dinucleotide, producing a number of intermediary metabolites, including 3-hydroxy kynurenine and quinolinic acid, which are involved in the neurodegenerative mechanisms that underlie Alzheimer’s disease (AD). Indolamine 2,3-dioxygenase (IDO), the first and rate-limiting enzyme of this pathway, is increased in AD, and it has been hypothesized that blocking this enzyme may slow the progression of AD. In this study, we treated male and female 3xTg-AD and wild-type mice with the novel IDO inhibitor DWG-1036 (80 mg/kg) or vehicle (distilled water) from 2 to 6 months of age and then tested them in a battery of behavioral tests that measured spatial learning and memory (Barnes maze), working memory (trace fear conditioning), motor coordination and learning (rotarod), anxiety (elevated plus maze), and depression (tail suspension test). The 3xTg-AD mice treated with DWG-1036 showed better memory in the trace fear conditioning task and significant improvements in learning but poorer spatial memory in the Barnes maze. DWG-1036 treatment also ameliorated the behaviors associated with increased anxiety in the elevated plus maze and depression-like behaviors in the tail suspension test in 3xTg-AD mice. However, the effects of DWG-1036 treatment on the behavioral tasks were variable, and sex differences were apparent. In addition, high doses of DWG-1036 resulted in reduced body weight, particularly in females. Taken together, our results suggest that the kynurenine pathway is a promising target for treating AD, but more work is needed to determine the effective compounds, examine sex differences, and understand the side effects of the compounds.

## Introduction

Alzheimer’s disease (AD) is a progressive neurodegenerative disorder that results in synaptic dysfunction and cerebral atrophy (Marcello et al., 2012; Pini et al., 2016). Behavioral consequences of AD include memory loss and dementia, accompanied by motor deficits and mood disorders, such as anxiety and depression (Lyketsos et al., 2011; Albers et al., 2015; Scheltens et al., 2016). Although the exact causes of AD are unknown, amyloid beta (Aβ) plaques and neurofibrillary tangles of tau protein have been identified as the neuropathological hallmarks of the disease (Glenner and Wong 1984; Stelzmann et al., 1995; Chong et al., 2018). The amyloid cascade hypothesis postulates that increased Aβ_42_ in the brain initiates a cascade of neurological deficits that cause the cognitive and behavioral symptoms associated with AD (Hardy and Allsop, 1991; Selkoe, 1991; Selkoe and Hardy, 2016). Oxidative stress, immune deficiencies, and glial dysfunction may also contribute to the progression of AD (Gella and Durany, 2009; Zhao et al., 2013; Heppner et al., 2015; Jain et al., 2015). However, the relationship between these proposed disease mechanisms, their temporal pattern of development, and their contributions to specific neurobehavioral deficits remain unclear.

Treatments for AD have focused on preventing the loss of acetylcholine and glutamate neurotransmitter functions (Francis, 2005; Kandimalla and Reddy, 2017). The cholinergic theory is based on the early degeneration of forebrain cholinergic neurons in AD patients, resulting in decreased acetylcholine levels at synapses (Francis et al., 1999). Drugs such as donepezil and galantamine function as cholinesterase inhibitors to decrease acetylcholine breakdown (Birks, 2006). Memantine targets glutamatergic transmission by blocking NMDA receptors to decrease excitotoxicity (Molinuevo et al., 2005; Olivares et al., 2012). Other neurotransmitter systems such as dopamine (Martorana and Koch, 2014; Nobili et al., 2017) and serotonin (Li et al., 2017; Vakalopoulos, 2017) may be involved in the progression of AD. There is also growing evidence that tryptophan is involved in the pathogenesis of AD independently of its role as the serotonin precursor.

**Figure 1:**
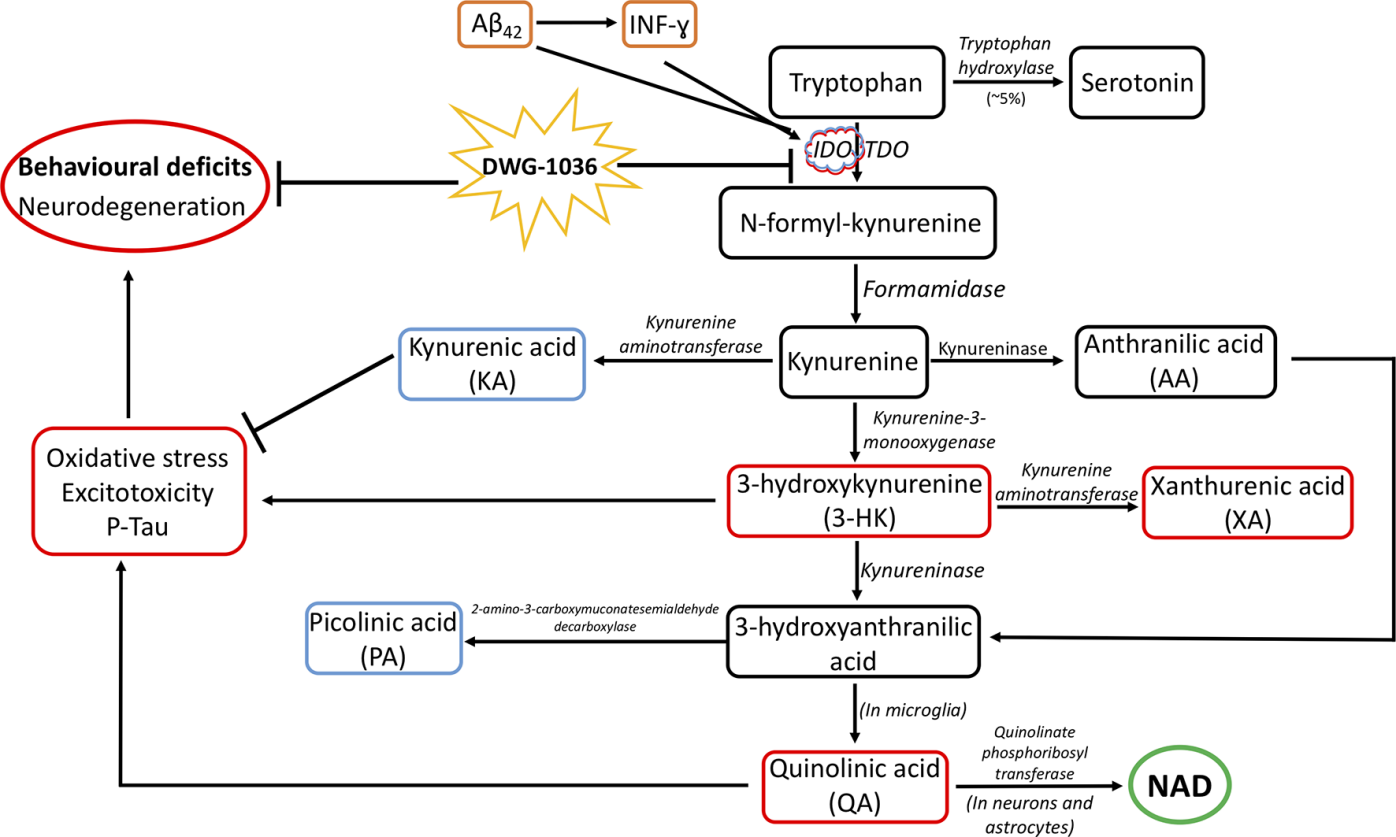
The kynurenine pathway (KP) and its suggested role in Alzheimer’s disease (AD) pathogenesis. Although 5% of tryptophan is converted to serotonin by tryptophan hydroxylase, the remainder gets metabolized by the KP. Indoleamine 2,3-dioxygenase (IDO) and tryptophan 2,3-dioxygenase (TDO) are the rate-limiting enzymes of the KP, which metabolize tryptophan to N-formyl-kynurenine, which is further metabolized into kynurenine, 3-hydroxykynurenine (3-HK), 3-hydroxyanthranilic acid, quinolinic acid (QA), and eventually, nicotinamide adenine dinucleotide (NAD). Kynurenine is converted to anthranilic acid and kynurenic acid, which is neuroprotective. 3-Hydroxykynurenine is neurotoxic and can be converted to xanthurenic acid. 3-Hydroxyanthranilic acid is converted to picolinic acid, which is neuroprotective, whereas QA is neurotoxic (Chen and Guillemin, 2010; Schwarcz and Stone, 2017). The involvement of the KP in AD pathogenesis is caused by the elevation of IDO by amyloid beta 42 (Aβ_42_) and interferon gamma (INF-ɣ), resulting in neurodegeneration and the cognitive and behavioral deficits shown in AD. It is proposed that the IDO inhibitor DWG-1036 will act to reduce the neurobehavioral deficits seen in AD. RED = neurodegenerative processes; BLUE = neuroprotective processes.

Tryptophan is involved in neurotransmission, immune function, and kynurenine synthesis (Moffett and Namboodiri, 2003; Ruddick et al., 2006), and 95% of the tryptophan in the body is metabolized through the kynurenine pathway (KP; Soliman et al., 2010). As shown in **Figure 1**, a cascade of enzymes converts tryptophan to nicotinamide adenine dinucleotide (NAD). Although the activity of the KP is necessary for tryptophan homeostasis, immune system regulation, and NAD synthesis, overactivity of the KP is associated with neurodegenerative disorders, including Parkinson’s disease (Havelund et al., 2017), Huntington’s disease (Mazarei and Leavitt, 2015), multiple sclerosis (Rejdak et al., 2002), amyotrophic lateral sclerosis (Guillemin et al., 2005b), and AD (Widner et al., 2000; Bonda et al., 2010; Gulaj et al., 2010; Campbell et al., 2014; Maddison and Giorgini, 2015). Research on the relationship between the KP and neurodegenerative diseases has focused on the neuroactive metabolites of tryptophan catabolism (Schwarcz and Stone, 2017). Of these, 3-hydroxy kynurenine (3-HK) and quinolinic acid (QA) are neurotoxic, whereas kynurenic acid (KA) and picolinic acid (PA) are protective (Urenjak and Obrenovitch, 2000; Lovelace et al., 2017).

Indoleamine 2,3-dioxygenase (IDO) and tryptophan 2,3-dioxygenase (TDO) are the first and rate-limiting enzymes of the KP (Munn et al., 1998; Moon et al., 2015) and are responsible for metabolizing tryptophan into N-formyl-kynurenine, which is broken down to form 3-HK and QA, the two neurotoxic metabolites of the KP (Chen and Guillemin, 2010). 3-HK increases oxidative stress (Mackay et al., 2006), which can exacerbate neurodegeneration and contribute to AD pathogenesis (Zhao et al., 2013; Wang et al., 2014). QA, which is only synthesized by microglia in the brain, is an N-methyl-D-aspartate (NMDA) agonist that can increase excitotoxicity (de Carvalho et al., 1996; Guillemin, 2012). Because QA is also involved in tau phosphorylation (Crowley et al., 1992; Rahman et al., 2009), it could increase neuronal and synaptic dysfunction (Dubal, 2018; Tracy and Gan, 2018). The neuroprotective properties of KA are caused by its function as an antagonist at NMDA receptors (Albuquerque and Schwarcz, 2013), hence it can decrease the excitotoxicity caused by QA. KA is also an antagonist at alpha-7 (α7) nicotinic receptors, reducing the endocytosis of Aβ_42_ (Nagele et al., 2002; Hernandez and Dineley, 2012). However, significantly less KA is produced relative to 3-HK and QA (Moroni et al., 2007). PA has protective effects because it enhances immune function (Grant et al., 2009).

IDO gene expression is stimulated by interferon gamma (INF-ɣ, Taylor and Feng, 1991; Jurgens et al., 2009) and by Aβ_42_ (Guillemin et al., 2003). IDO levels are increased in the hippocampus of AD patients (Guillemin et al., 2005a), and INF-ɣ and IDO levels are increased in the cerebrum of female triple transgenic mice (3xTg-AD), a commonly used model of AD (Fertan et al., 2019a). Thus, overactivity of IDO in the KP may integrate the various mechanisms involved in the pathogenesis of AD, leading to neuronal loss and behavioral deficits. These include increases in Aβ_42_ levels, tau phosphorylation, immune dysfunction, and oxidative stress. On the other hand, the role of TDO in AD pathogenesis is unclear. Unlike the universal expression profile of IDO, TDO is mostly found in the liver (Dale et al., 2000), yet it has been measured in the frontal cortex of individuals with schizophrenia (Miller et al., 2004) and mouse brains at different levels during development (Kanai et al., 2009). Unlike IDO, TDO has not been shown to be regulated by inflammatory cytokines or Aβ42; however, the expression levels are increased by glucocorticoids (Green et al., 1975; Nakamura et al., 1987). Wu et al. (2013) showed significantly elevated levels of TDO in the cerebellum, but not cerebrum, of 3xTg-AD mice and hippocampi of humans with AD. Because 3-HK is increased in the serum (Schwarz et al., 2013) and QA is increased in the hippocampus of AD patients (Guillemin et al., 2005a) as well as 3xTg-AD mice (Fertan et al., 2019a), the KP may be a worthy target for AD treatment (**Figure 1**). There is evidence that reducing KP activity can ameliorate some of the symptoms of AD in animal models (Vamos et al., 2009; Zwilling et al., 2011; Yu et al., 2014; Deora et al., 2017).

In the current study, we tested the ability of a novel inhibitor of IDO and TDO (DWG-1036) to reverse the behavioral deficits seen in the 3xTg-AD mice. These mice were engineered by injecting *APP_Swe_* and *tau_P301L_* transgenes into single-cell embryos of homozygous *PS1_M146V_* knock-in mice, causing an increase in Aβ_42_ and tau phosphorylation (Oddo et al., 2003b). Interneuronal amyloid aggregation in the frontal cortex and the hippocampus starts around 2 months of age in these mice, and plaque accumulation accompanied by neuroinflammation is observed at 6 months of age, with tau tangles at 12 months of age (Oddo et al., 2003a; Belfiore et al., 2019). Working memory deficits in the 3xTg-AD mice in the eight-arm radial maze have been shown at 2 months of age (Stevens and Brown, 2015), and spatial memory deficits in the Barnes maze have been shown at 6 months of age (Stover et al., 2015a, Stover et al., 2015b). Increased anxiety and depression-like behaviors have also been shown in the 3xTg-AD mice by 7 months of age (Romano et al., 2014; Zhang et al., 2016; Nie et al., 2017). However, these mice show better motor performance on the rotarod than wild-type (WT) controls (Stover et al., 2015a; Garvock-de Montbrun et al., 2019). In this study, male and female 3xTg-AD and WT mice were treated with DWG-1036 from 2 to 6 months of age and then tested in a behavioral test battery that measured spatial learning and memory, working memory, motor coordination and learning, anxiety, and depression. We hypothesized that DWG-1036 would decrease or reverse the behavioral deficits observed in the 3xTg-AD mice.

## Methods

### DWG-1036 Synthesis and Pharmacokinetics

**Figure 2:**
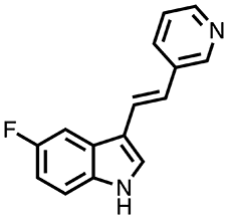
The molecular structure of DWG-1036. The chemical formula of the compound is C_15_H_11_FN_2_, and the molecular weight is 238.265 g/mol.

DWG-1036 (C_15_H_11_FN_2_) is a synthetic IDO inhibitor (**Figure 2**), developed by Dr. Donald F. Weaver’s group at the Krembil Research Institute in Toronto, Canada, using the following procedure: A 500 mL round-bottom flask was charged with the 3-pyridylacetic acid (HCl salt, 13.7 g, 78 mmol), then anhydrous 1,4-dioxane (200 mL) was added. Triethylamine (28 mL, 200 mmol) was added dropwise at room temperature, and the mixture was stirred for 30 min. 5-Fluoroindole-3-carboxaldehyde (10.9 g, 66.8 mmol) was then added, followed by piperidine (14.5 mL, 147 mmol). The reaction mixture was heated at 110°C for 18 h. The reaction mixture was cooled to room temperature, and an additional aliquot of 3-pyridyl-acetic acid (HCl salt, 2.32 g, 13.4 mmol) and piperidine (1.3 mL, 13.4 mmol) was added. The reaction mixture was heated at 110°C for an additional 18 h. The reaction mixture was cooled to room temperature and then partitioned between aqueous ammonium chloride (100 mL) and ethyl acetate (200 mL). The organic fraction was separated and then washed with brine (50 mL) and dried with anhydrous Na_2_SO_4_. After filtration, silica gel was added, and the solution was concentrated *in vacuo*. Automated flash column chromatography (100:0 to 1:1 hexanes:ethyl acetate gradient) afforded 12.5 g of the desired product. The free base was suspended in water (1 mL per 20 mg of compound); 5% aqueous HCl was added slowly until most of the suspended material had dissolved. The reaction mixture was filtered and then concentrated *via* lyophilization to provide 11.3 g of the desired product as a bright yellow solid (HCl salt, 62% yield). HPLC purity analysis was carried out using a Waters 1525EF Binary pump system equipped with a dual wavelength absorbance detector (254 nm, 280 nm) and a manual injector. The stationary phase consisted of a Silicycle Silia Chrom SB C18 column (250 × 4.6 mm), and the mobile phase used water (0.1% trifluoroacetic acid) and acetonitrile (0.1% trifluoroacetic acid) at the following gradient system, eluting at 1 mL/min: 80% H_2_O/20% CH_3_CN for 1 min, then a linear ramp to 5% H_2_O/95% CH_3_CN over 7 min, hold at 5% H_2_O/95% CH_3_CN for 4 min, and then return to a linear ramp to 80% H_2_O/20% CH_3_CN for 3 min.

The IC_50_ value for IDO was determined to be 80 μM using the enzymatic *in vitro* assay of Takikawa et al. (1988) and 7 μM in a cell-based assay. The IC_50_ value of DWG-1036 for TDO was determined as 9.7 μM. As measured in pilot studies (data not shown), DWG-1036 was detectable (μ = 59, 198 ng/mL) in the brain of the WT (B6129SF2/J) mice 15 min after administration *via *oral gavage and reached the highest levels (μ = 69,753) in 30 min. The half-life of DWG-1036 was calculated as 1.24 h, the area under the curve (AUC) from 0 to last measured point (AUC0–last) was 138,652 ng.h/mL. The AUC for 0 to infinity was (AUC0-∞) 138,760 ng.h/mL, and the mean residence time (MRT) was 1.79 h.

### DWG-1036 Tolerability Testing

To determine any side effects or dose by genotype interactions of DWG-1036, a tolerability study was performed prior to treatment and behavioral testing. Eight wild-type and two 3xTg-AD mice at 2 months of age were treated with distilled water (vehicle) or 30, 60, or 80 mg/kg of DWG-1036 once a day or with 80 mg/kg of DWG-1036 twice a day for 25 days. Body weights of the mice were recorded every day before treatment and compared with a generalized linear mixed model regression analysis. All of the procedures and experimental techniques used in the tolerability study were approved by the Dalhousie University Council of Animal Ethics (16-016).

**Figure 3:**
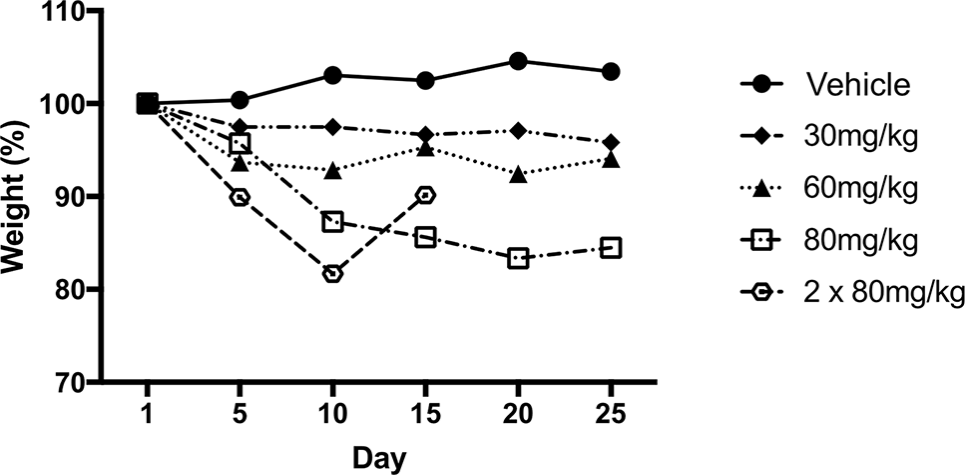
Change in body weight (%) of mice over 25 days of receiving vehicle or 30, 60, or 80 mg/kg DWG-1036 once a day or 2 × 80 mg/kg each day. Body weights on day 1 were normalized to 100%.

There were significant differences in body weight between treatment groups over days as the models including the effect of treatment type (AIC_Treatment_ = 728.64, LH = 18.625, p < 0.005), day (AIC_Day_ = 734.10, LH = 66.079, p < 0.005), and the treatment by day interaction (AIC_Treatment : Day_ = 757.57, LH = 226.85, p < 0.005) differed significantly from the null model (AIC_Null_ = 716.02). Although the weights of the mice receiving the vehicle increased over the 25-day period, mice receiving 30 or 60 mg/kg of DWG-1036 did not gain weight. Mice receiving 80 mg/kg showed weight loss over the treatment period, and the mice receiving 80 mg/kg twice a day had to be removed from the study by day 15 because of excessive weight loss (**Figure 3**). Based on these results, it was decided to use a dose of 80 mg/kg DWG-1036 once a day in the experiment.

### Subjects

The study began with 41 3xTg-AD (23 female/18 male) and 52 B6129SF2/J wild-type control mice (30 female/22 male). Because of removal from the study for excessive weight loss, 34 3xTg-AD (19 female/15 male) and 37 B6129SF2/J wild-type control mice (16 female/21 male) completed behavioral testing (**Table 1**). All of the mice were born in-house from breeding pairs originally purchased from Jackson Laboratories in Bar Harbor, Maine (JAX stock: 34830). After weaning, the mice were housed in same sex groups of two to four in translucent polyethylene cages (13 × 30 × 15 cm) with wire food hoppers and micro-isolator filter lids in a climate-controlled (20 ± 2℃) vivarium on a reversed 12:12 h light/dark cycle with lights off at 10:00 am. The mice were fed Purina Laboratory Rodent Chow #5001 (Agribrand Purina, Strathroy, Ontario, Canada) and tap water *ad libitum*. A black polyethylene tube (4 cm diameter, 7.5 cm length) was placed in the cages for environmental enrichment. Cages were cleaned once a week. All of the procedures and experimental techniques used in this study were approved by the Dalhousie University Council of Animal Ethics (16-017).

**Table 1:** Sample sizes for each group of mice.

B6129SF2/J (Wild type)		3xTg-AD	
DWG-1036	Vehicle	DWG-1036	Vehicle
11/11 males	11/11 males	8/10 males	7/8 males
5/19 females	10/11 females	11/15 females	8/8 females

The first number in each box represents the number of mice used in the behavioral tests. The second number indicates the starting sample sizes for each group. Due to excessive weight loss, some mice were removed from the study at different times during the treatment period.

### Treatment

Treatment with DWG-1036 or vehicle (distilled water) started at 2 months of age and continued until the end of behavioral testing. Once a day (around 5 pm, which was at least an hour after behavior testing during that period), mice were weighed and then given a dose of 80 mg/kg DWG-1036 with an injection volume of 0.01 ml/g by oral gavage using 1-ml syringes and 22-gauge gavage needles covered with a flavored (pomegranate and strawberry) edible lubricant (Sliquid Swirl^®^). If a mouse lost more than 2 grams in weight in a 24-h period, no treatment was applied. Mice not receiving treatment for 5 days in a row or 10 days in total were removed from the study.

### Behavioral Tests

Behavioral testing started when the mice were 6 months of age, and tests were given in the order listed below. Tests were conducted between 10.00 am and 4.00 pm each day in a specific test room. The experimenter was blind to the genotype and treatment condition of each mouse during behavioral testing.

#### Elevated Plus Maze

Mice were carried to a dark room, separated from the test room, in their home cages, with the water bottle removed. Then individual mice were carried to the elevated plus maze (EPM) in a clean plastic container while their cage mates remained in the holding room with *ad libitum* food and water. The EPM consisted of a plus-shaped maze with two open arms (30 × 5 cm) with a 4-mm lip to prevent the mouse from slipping off and two closed arms (30 × 5 cm) with transparent Plexiglas walls (15 cm high) located across from each other. The arms were connected by a center square (5 × 5 cm). The floor of the maze was black Plexiglas, and the walls of the closed arms were clear. Testing was completed in a room (2 × 5 m) illuminated by two 60-watt white light bulbs. Each mouse was tested on one 5-min trial, and between mice, the maze was cleaned with Sparkleen^®^ solution. At the beginning of the trial, the mice were placed in the center square. A camera 2.1 m above the maze recorded the movement of the mice throughout the trial. The time in the open and closed arms and the distance traveled were analyzed with a computerized tracking system (EthoVivion^Ⓡ^, Noldus, Wageningen, The Netherlands). The frequency of freezing (remaining completely immobile except for respiration) bouts was recorded using a computerized tracking system (Limelight^Ⓡ^, Actimetrics Inc., Wilmette, IL). According to O’Leary et al. (2013), the total distance traveled in the EPM is a measure of locomotion, whereas frequency of entering the closed arms and freezing are measures of anxiety.

#### Rotarod

The AccuRotor accelerating rotarod (Accuscan Instruments Inc., Columbus, Ohio), consisted of a 44-cm-long acrylic rod with a diameter of 3 cm, covered with rubber to provide better gripping. The rod was separated into four 11-cm sections by circular Plexiglas dividers (15 cm high), allowing four mice to be tested concurrently. There were separate holding chambers 39 cm beneath each section of the rod. The latency to fall from the rod was measured with electronic timers, which automatically stopped when the mouse touched the surface of the holding chamber. The rotarod was located in a 112 × 260 cm room, lit by a single 60-watt red light. Mice were gently held by their tails and placed on the floor of the holding chamber. Once all four mice were in the rotarod, they were placed on the rod, facing the opposite direction of the rotation as well as the experimenter. The maximum length of each trial was 360 s, and during the trial, the rod gradually accelerated from 0 to 48 rotations per min. After the last mouse fell from the rod, a 1-min break was given before starting the next trial. Mice completed six trials on the rotarod per day for 5 consecutive days. The rotarod was cleaned with soap and water after each group of mice completed a daily test session of six trials. The time to fall from the rotarod is a measure of motor coordination and learning (O’Leary et al., 2018).

#### Barnes Maze

The Barnes maze (BM) was a white polyethylene platform (122 cm diameter) elevated 48.4 cm from the floor with 16 holes (4.45 cm diameter) equally spaced around the perimeter 1.3 cm from the edge (O’Leary and Brown, 2012). Four of the holes (4, 8, 12, and 16) were capable of having a black plastic escape box beneath them. A buzzer (0–37.2 kHz, 89 dB) and two 150-watt flood lamps placed 155 cm above the maze were used as aversive stimuli. A polyvinyl-chloride tube (8 cm diameter, 12.5 cm height) was used to hold the mouse in the center of the maze until the trial began. A camera was mounted 1.7 m above the maze to record the trials. Mice were tested in groups of three to five, and each mouse in the group was assigned a specific escape hole location. There were five phases in the test procedure: habituation, acquisition training, acquisition probe, reversal training, and reversal probe (O’Leary and Brown, 2013). During the habituation phase, mice were placed in a 2-L glass beaker, which was inverted over the assigned escape hole. The mice were then free to explore the escape hole, escape box, and the adjacent area for 2 min. The acquisition training phase consisted of two trials per day for 15 days. On each trial, mice were placed in the center tube and after an interval of 5–10 s, the tube was lifted, and the buzzer was turned on. The mice were given 300 s to locate the escape hole, and if they did not enter the escape box within this time, they were led to the escape hole with a plastic cup that was used to transport the mice. The maze was cleaned between trials with Sparkleen^®^ solution to prevent odor cues from developing around the escape holes. The measures of learning (latency to enter the escape hole, distance traveled, and average speed) were analyzed for each trial using Ethovision^®^ (Noldus, Wageningen, The Netherlands). The number of errors (when a mouse dips its head into a hole that is not the escape hole) and correct head dips were recorded by the experimenter. Repeated head dips into the same hole were recorded as one head dip. The maze was rotated 90° between each group, and all escape holes and the escape box were cleaned to prevent the use of extraneous cues.

The day after acquisition training was completed, the mice were given a 5-min memory probe trial with the buzzer turned off. During this trial, the escape box was removed, and the maze was rotated 45° so that a non-escape hole was in the correct escape hole location. For analysis of spatial memory, the maze was divided into 16 pie-shaped zones, and the number of entries and time spent in each zone were recorded. The mice were then given a 5-day reversal training phase with the escape hole moved to the opposite side of the maze followed by a reversal probe trial using the same procedure as during the acquisition probe trial. Measures of learning and memory were analyzed for the reversal test.

#### Tail Suspension Test

The tail suspension test (Med Associates, St. Albans, VT, USA) consisted of a box (32 × 33 × 33 cm) that was open on one side to allow an observer to view the subjects and for video recording. An aluminum strip (11.5 × 2.2 × 0.15 cm) was suspended vertically from a strain gauge within the enclosure, which the mouse was attached to by its tail with duct tape. After their weights were recorded, individual mice were placed on an upside-down cage located under the aluminum strip, and their tails were gently attached to the strip. Then the cage was slowly removed to allow the mice to hang from the strip by its tail. Mice were observed for immobility for one 6-min trial, which was recorded with a video camera. Testing was done in a quiet room lit by two 60-watt white light bulbs. At the end of the trial, the empty cage was placed under the mouse so it could stand on four feet without any pressure on the tail. Then the duct tape was removed to free the tail, and the mouse was carried back to its home cage. During the trial, frequency of immobility (lack of escape attempts) was analyzed as a measure of depression-like behavior (Can et al., 2012).

#### Trace Fear Conditioning

Trace fear conditioning and testing took place in two identical MED Associates Inc. (St. Albans, VT) fear conditioning chambers. The front, top, and back of the chamber were transparent Plexiglas, and the other two remaining were stainless steel. The floor of the chamber consisted of 36 3.2-mm stainless steel rods that were capable of delivering an electric shock. A speaker was attached to one of the stainless steel walls, and a video camera was mounted in front of one of the Plexiglas walls to record the behavior of the mouse. The procedure consisted of a training and test phase, which took place on 2 consecutive days. During the training phase, mice were placed in the chamber, and their levels of baseline freezing were recorded for 774 s. During this time, five 80-dB tone cues lasting 15 s were presented with 130-s intervals. Each tone cue was followed by a 1-s 0.7-mA foot shock, delivered 30 s after the tone. Thirty seconds after the last shock, the mice were removed from the chamber and returned to their home cage, and the chamber was cleaned with Sparkleen^®^ solution.

In the working memory test phase, mice were placed in the second chamber, a modified version of the chamber used during training, for 265 s. Black Plexiglas was placed over the floor of the chamber to cover the steel rods, the inside walls of the testing chamber were covered with black and white striped plastic, and a novel lemon odor was introduced into the chamber. The mice were then placed in this modified chamber, and their freezing time was recorded for 2 min, followed by a 15-s-long 80-dB tone identical to the one presented during training. After the tone, the duration of freezing was recorded for another 130 s as a measure of working memory (Gilmartin and Helmstetter, 2010; Raybuck and Lattal, 2014).

### Statistical Analyses

Analysis of variance (ANOVA), generalized linear mixed model regressions, and chi-square tests were used to analyze the data. To deal with unequal sample sizes, a Type 2 calculation of sums of squares was used. Differences between individual groups were determined using 95% confidence intervals and indicated in the graphs using asterisks. “R: The R Project Statistical Computing^®^” version 3.5.2 (2018-12-20) - “Eggshell Igloo” was used for all of the statistical analyses, and the graphs were generated in “Graph Pad Prism VII^®^” using group means and standard errors (SEM). Data from each test were first analyzed for the existence of a sex difference. If there was a significant sex difference, the data were analyzed separately for each sex. If there was no significant sex difference, data were analyzed by pooling the sexes. Based on this criterion, sex differences were found in the Barnes maze probe trial total distance, freezing duration in trace fear conditioning, latency to fall from the rotarod, and freezing frequency in the elevated plus maze.

## Results

### Mice Removed From the Study

Over the 4-month treatment period, 22 mice were removed from the study for showing significant weight loss (**Table 1**). The removal rate differed significantly between the groups (χ²(7) = 37.62, p < 0.001). Only two mice receiving the vehicle lost weight during the treatment period, whereas 20 mice receiving DWG-1036 lost weight, 18 of which were female. Thus, there was a sex difference in the side effects of the treatment.

### Elevated Plus Maze

#### Locomotion

**Figure 4:**
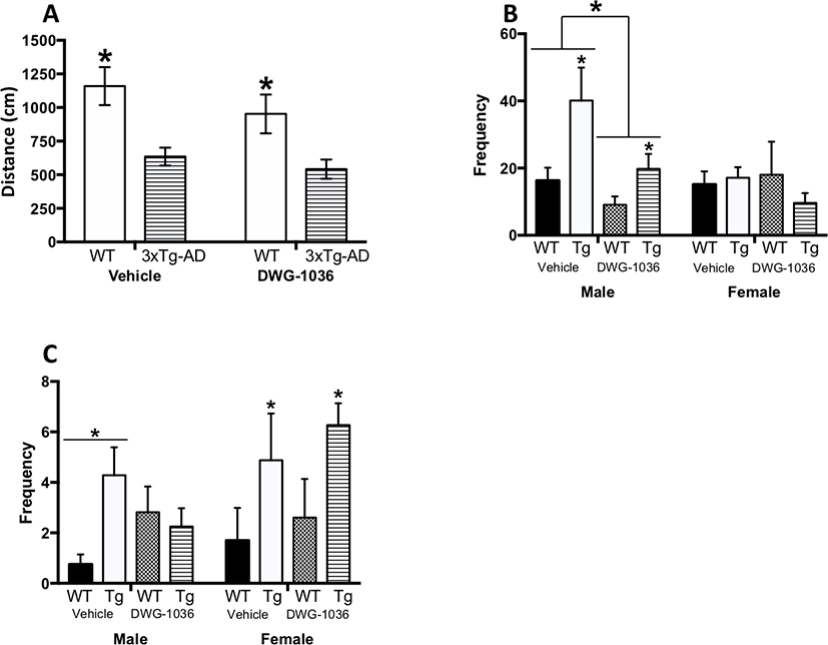
Performance of male and female wild-type (WT) and 3xTg-AD mice given vehicle or DWG-1036 on the elevated plus maze. **(A)** Mean (±SEM) distance (in centimeters) traveled by the mice, a measure of locomotor behavior. **(B)** Mean (+SEM) frequency of visiting the open arms, a measure of reduced anxiety. **(C)** Mean (+SEM) frequency of freezing behavior, a measure of anxiety. *’s are used to present individual group differences determined by 95% confidence intervals.

The 3xTg-AD mice traveled a less distance on the EPM than WT mice (F(1, 67) = 17.469, p < 0.001), but there were no main effects of treatment (F(1, 67) = 1.679, p > 0.05) nor any significant interactions (all p > 0.05, **Figure 4A**).

#### Open Arm Entry

The 3xTg-AD mice visited the open arms more than the WT mice (F(1, 63) = 5.214, p < 0.05), and this was decreased by DWG-1036 treatment, but not significantly (F(1, 63) = 3.058, p = 0.09). Overall, DWG-1036-treated mice visited the open arms less than the controls (F(1, 63) = 6.614, p < 0.05). There was also a significant genotype by sex interaction (F(1, 63) = 9.063, p < 0.01) because the female 3xTg-AD mice had lower frequencies of open arm entry than the female WT mice; however, the male 3xTg-AD mice had higher frequencies compared to the male WT mice (**Figure 4B**).

#### Freezing Frequency

For the male mice, there were no main effects of genotype (F(1,34) = 2.584, p = 0.12) or treatment condition (F(1,34) = 0.266, p = 0.61), but there was a genotype by treatment interaction (F(1, 34) = 6.030, p < 0.05), as the freezing frequency was decreased in 3xTg-AD mice treated with DWG-1036 and increased in the WT controls (**Figure 4C**).

For the females, there was a genotype difference (F(1,30) = 5.946, p < 0.05) because the 3xTg-AD mice froze more frequently than the WT control mice, but there was no difference between the treatment conditions (F(1,30) = 0.739, p = 0.40, **Figure 4C**).

#### Rotarod

**Figure 5:**
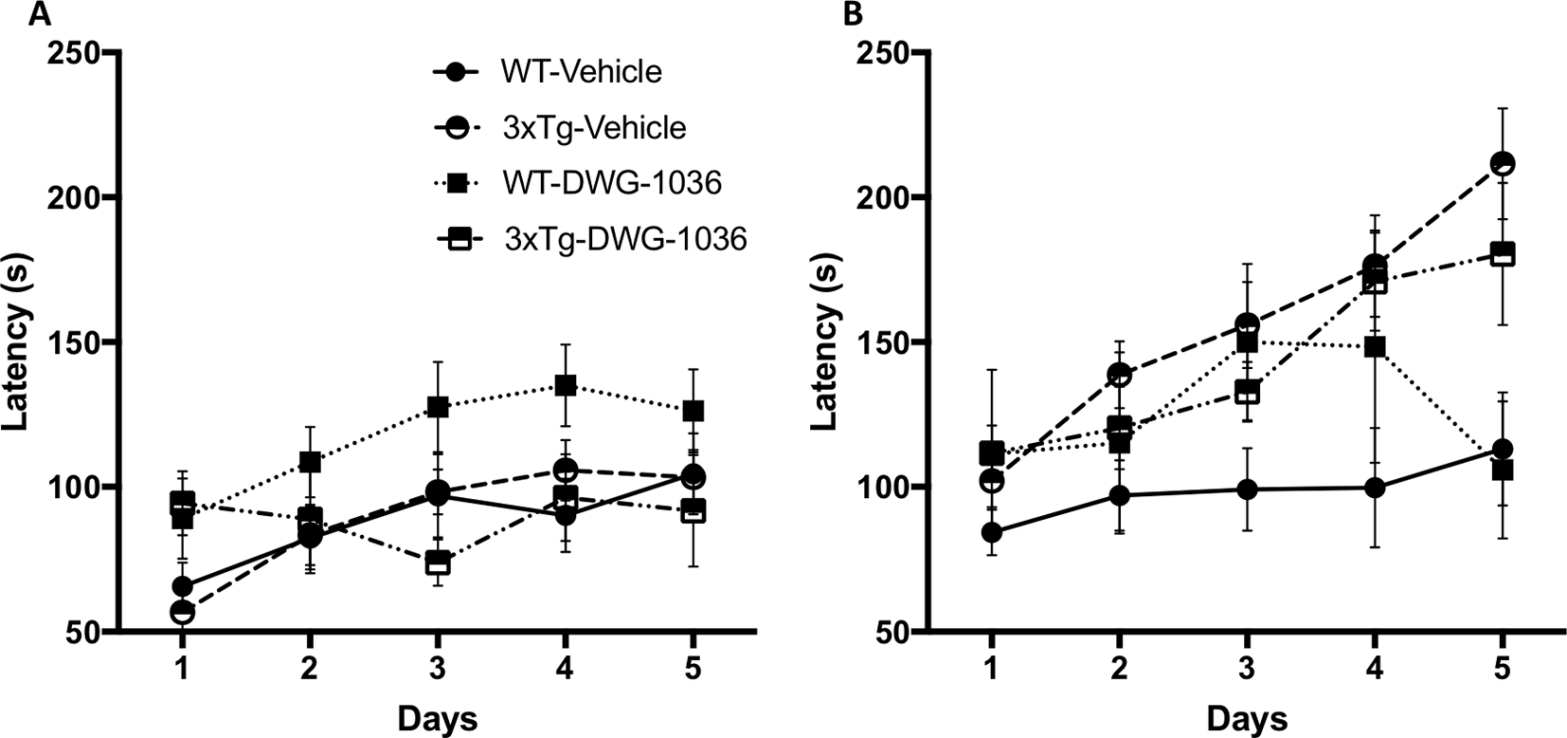
Mean ( ± SEM) latency (in seconds) to fall from the rotarod on each of the days for **(A)** male and **(B)** female mice in each treatment group. The rotarod was used to measure motor learning and coordination.

For the male mice, models including test day (AIC_Day_ = 1632.7, LH = 39.95, p < 0.005) and genotype by treatment by day interaction (AIC_Genotype : Treatment:Day_ = 1609.7, LH = 12.06, p < 0.05) differed significantly from the null model (AIC_null_ = 1600.7). Although all of the mice improved over the 5-day test period, the DWG-1036-treated male WT mice showed the greatest improvement (**Figure 5A**).

For the female mice, models including test day (AIC_Day_ = 1759.1, LH = 49.26, p < 0.005), genotype (AIC_Genotype_ = 1722.2, LH = 6.35, p < 0.05), and genotype by day interaction (AIC_Genotype : Day_ = 1721.5, LH = 28.91, p < 0.005) differed significantly from the null model (AIC_null_ = 1717.8). The 3xTg-AD females had longer latencies to fall from the rod compared to the WT mice, and they improved more over the 5-day period (**Figure 5B**). There were no significant drug treatment effects or interactions

### Barnes Maze

#### Acquisition Latency

**Figure 6:**
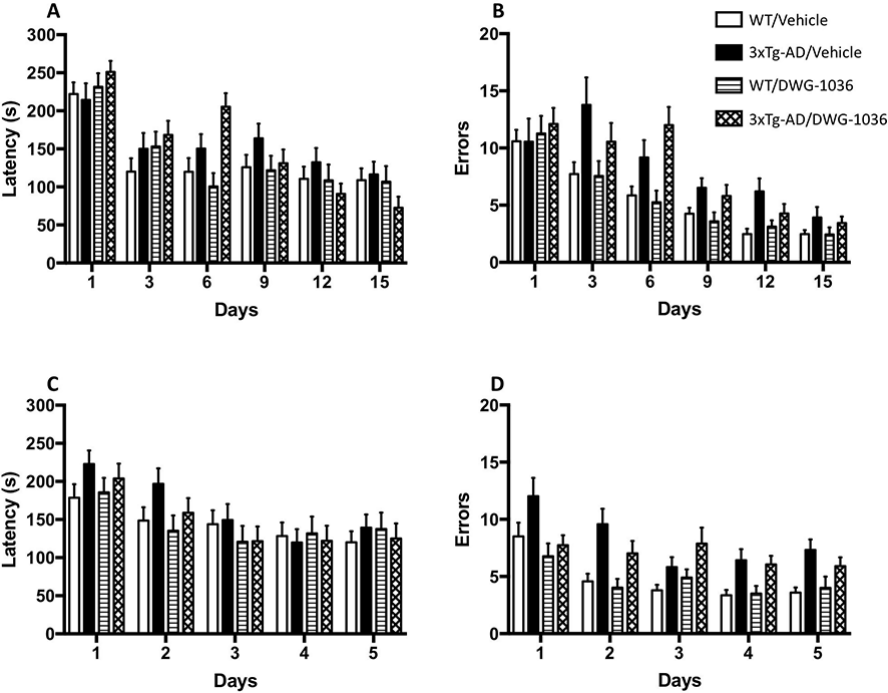
Learning performance of mice in each group on the acquisition and reversal phases of the Barnes maze. Mean ( + SEM) latency (in seconds) for male and female 3xTg-AD and wild-type (WT) control mice to reach the goal box in the acquisition **(A)** and reversal **(C)** test days. Mean ( + SEM) frequency of errors for male and female 3xTg-AD and WT control mice to reach the goal box in the acquisition **(B)** and reversal **(D)** test days.

Models including the effect of day (AIC_Day_ = 25448, LH = 327.89, p < 0.005), the genotype by day interaction (AIC_Genotype:Day_ = 25151, LH = 44.614, p < 0.005), and the genotype by treatment by day interaction (AIC_Genotype:Treatment:Day_ = 25135, LH = 27.397, p < 0.05) differed significantly from the null model (AIC_null_ = 25148). Overall, all mice reduced their latencies to find the escape hole over the 15-day period, and the 3xTg-AD mice treated with DWG-1036 showed a greater reduction than the other groups (**Figures 6A**).

#### Acquisition Errors

Models including day (AIC_Day_ = 14525, LH = 383.81, p < 0.005), genotype (AIC_Genotype_ = 14178, LH = 14.94, p < 0.005), and a genotype by day interaction (AIC_Genotype:Day_ = 14181, LH = 31.261, p = 0.005) differed significantly from the null model (AIC_null_ = 14169). Although all mice decreased their number of errors over the 15-day period, the decrease was greater for the WT mice than the 3xTg-AD mice, and there was no significant effect of DWG-1036 (**Figures 6B**).

#### Reversal Latency

The models including day (AIC_Day_ = 8122.8, LH = 88.254, p < 0.005) and genotype by day interaction (AIC_Genotype:Day_ = 8047.6, LH = 10.763, p < 0.05) differed from the null model significantly (AIC_null_ = 8042.6). Although all of the mice improved in the 5-day period, the 3xTg-AD mice improved more than the WT controls, but neither group showed a treatment effect (**Figures 6C**).

#### Reversal Errors

The models including day (AIC_Day_ = 4317.1, LH = 52.719, p < 0.005), genotype (AIC_Genotype_ = 4289.1, LH = 18.711, p < 0.005), and treatment by day interaction (AIC_Treatment : Day_ = 4078.0, LH = 15.084, p < 0.005) differed significantly from the null model (AIC_null_ = 4272.4). Although all of the mice decreased their number of errors over the 5-day period, the WT mice made fewer errors than the 3xTg-AD mice. Moreover, the DWG-1036-treated mice improved less than the vehicle treated mice, regardless of genotype (**Figures 6D**).

#### Acquisition Probe

**Figure 7:**
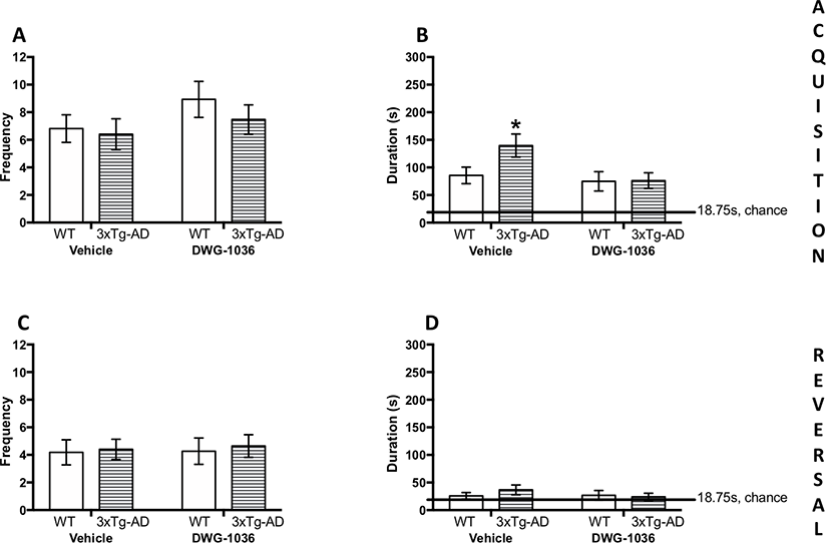
Mean (±SEM) frequency of entering the correct sector (1/16 of the maze) in the acquisition **(A)** and reversal **(C)** memory probe trials in the Barnes maze. Mean (±SEM) time (in seconds) spent in the correct sector (1/16 of the maze) in the acquisition **(B)** and reversal **(D)** memory probe trials in the Barnes maze. *’s are used to present individual group differences determined by 95% confidence intervals.

For the frequency of visiting the correct hole, there were no main effects of genotype, treatment, or sex, nor any significant interactions (all p > 0.05, **Figure 7A**). For the time spent by the correct hole, the 3xTg-AD mice spent more time by the correct hole than the WT mice; however, the difference was not significant (F(1, 66) = 2.921, p = 0.09). On the other hand, the DWG-1036-treated mice spent significantly less time by the correct hole than the vehicle-treated mice (F(1, 66) = 4.348, p < 0.05, **Figures 7B**).

#### Reversal Probe

For the time spent by the correct hole and the number of times visiting the correct hole, there were no main effects of genotype, treatment, or sex, nor any significant interactions (all p > 0.05, **Figure 7C, D**).

### Tail Suspension

**Figure 8:**
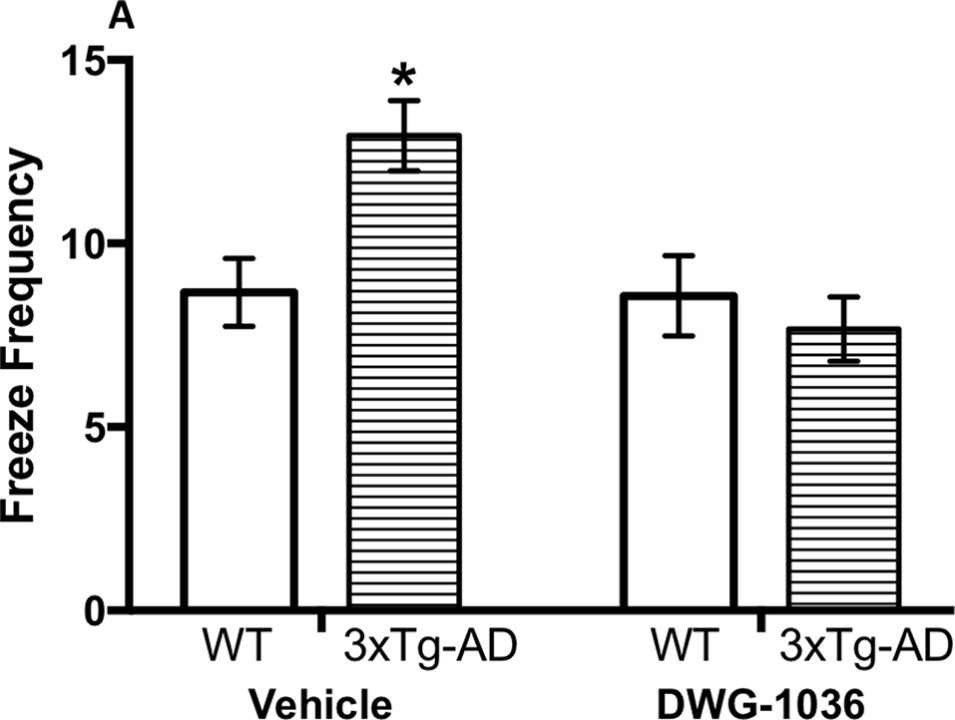
Mean ( ± SEM) frequency of freezing in the tail suspension test in mice of each group, a measure of depression-like behavior. *’s are used to present individual group differences determined by 95% confidence intervals.

Although the 3xTg-AD mice showed a higher immobility frequency compared to the WT mice, the difference was not statistically significant (F(1, 62) = 3.774, p = 0.057). However, there was a significant effect of treatment (F(1, 62) = 6.360, p < 0.05) because the vehicle-treated mice were immobile more frequently overall compared to the DWG-1036-treated mice. There was also a genotype by treatment interaction (F(1, 62) = 7.011, p < 0.05): the DWG-1036-treated 3xTg-AD mice stayed immobile less than the vehicle-treated 3xTg-AD mice, but there was no such difference for the WT mice (**Figure 8**).

### Trace Fear Conditioning

**Figure 9:**
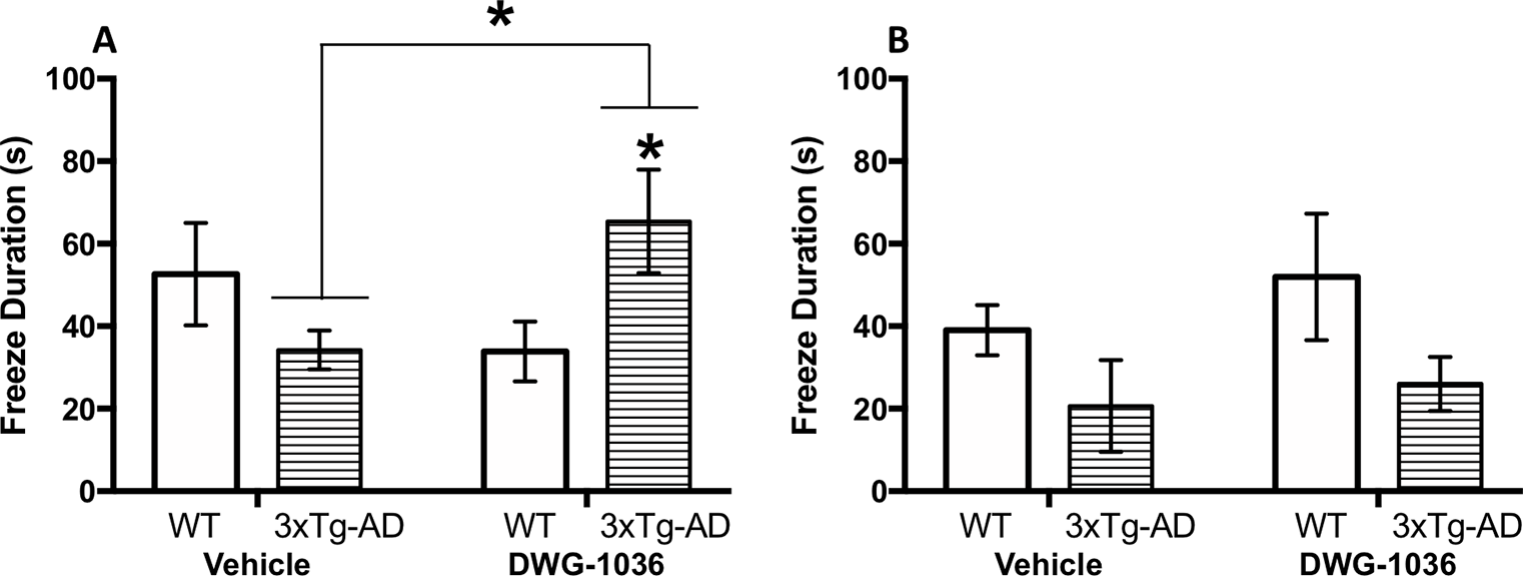
Mean ( ± SEM) duration of freezing by **(A)** males and **(B)** females in the working memory test of the trace fear conditioning procedure. *’s are used to present individual group differences determined by 95% confidence intervals.

The data for freeze duration were analyzed by calculating the difference between the duration of freezing before and after the sound cue and comparing these values between the groups. For the male mice, there were no main effects of genotype (F(1,27) = 0.123, p = 0.73) or treatment (F(1,27) = 0.007, p = 0.93), but there was a significant interaction between these factors (F(1,27 = 4.820, p < 0.05): although the DWG-1036-treated 3xTg-AD mice froze longer than the vehicle-treated 3xTg-AD mice, the opposite was the case for the WT control mice (**Figure 9A**).

For the female mice, there was a significant effect of genotype (F(1,26) = 4.950, p < 0.05) because the WT mice froze longer than the 3xTg-AD mice. On the other hand, treatment condition had no effect (F(1,26) = 0.700, p = 0.41, **Figure 9B**).

## Discussion

The 3xTg-AD mouse model has deficits in spatial and working memory but shows improved motor coordination and motor learning compared to the WT mice. The 3xTg-AD mice also show increased anxiety and depression-like behaviors. The aim of this study was to determine if the novel IDO/TDO inhibitor DWG-1036 reversed these deficits.

### Cognitive Function

In this experiment, cognitive function was measured in tests of spatial learning and memory (Barnes maze) and working memory (trace fear conditioning). Stover et al. (2015b) showed deficits in learning in the Barnes maze at 6 months of age in 3xTg-AD mice. Our results agree with their findings because the 3xTg-AD mice made more errors than WT mice in the reversal phase. However, in the acquisition phase, the greatest improvement was shown by the 3xTg-AD mice treated with DWG-1036. This suggests that the hippocampal damage seen in the 3xTg-AD mice (Oddo et al., 2003b) can be, up to a degree, halted or reversed by blocking the KP. The activity of the KP in the hippocampus has been studied in various ways. Increased IDO activity and quinolinic acid levels have been measured in hippocampi of AD patients (Guillemin et al., 2005a). Similarly, decreased KA to QA and KA to 3-HK ratios have been shown in the hippocampi of patients with depression (Savitz et al., 2015), which contributes to episodic memory deficits (Young et al., 2016). Moreover, xanthurenic acid, another KP metabolite, has been shown to reduce excitatory postsynaptic potentials (EPSPs) in mouse hippocampal slices (Neale et al., 2013). Increased microglia activation is negatively correlated with hippocampal volume of AD patients (Femminella et al., 2016). Because QA is only synthesized in microglia and is involved in excitotoxicity and neuronal loss, it may be at least partly responsible for the hippocampal shrinkage in AD. Indeed, NMDA receptor antagonists, such as memantine, which is commonly used in AD treatment (Kishi et al., 2017), reduce quinolinic acid-induced hippocampal damage (Keilhoff and Wolf, 1992). However, because IDO-induced KP activity increases quinolinic acid production, treatments reducing IDO may be more specific and selective at reducing NMDA-induced excitotoxicity.

Deficits in working memory function have been shown in the 3xTg-AD mice as early as 2 months of age (Stevens and Brown, 2015; Fertan et al., 2019b). In the trace fear conditioning test in this study, female 3xTg-AD mice showed working memory deficits compared to female WT mice, but these were not reversed by DWG-1036 treatment. On the other hand, male 3xTg-AD mice treated with DWG-1036 showed improvements in working memory compared to those receiving the vehicle, suggesting a sex difference in DWG-1036 action. Similar sex differences have been shown in rats because increased KP metabolites contributed to memory deficits in male rats, but no differences were found in females or gonadectomized males (Baratta et al., 2018). Moreover, when injected in the striatum with quinolinic acid, male and ovariectomized female rats lost significant amounts of weight, whereas females with intact ovaries did not (Zubrycki et al., 1990). Together, these findings suggest that male and female gonadal hormones modulate the KP in different ways (de Bie et al., 2016). The brain areas involved in trace fear conditioning, such as the frontal cortex and the amygdala (Runyan et al., 2004; Gilmartin and Helmstetter, 2010; Song et al., 2015), are rich in sex hormone receptors (Cooke et al., 2003; Cushing et al., 2008; Zeidan et al., 2011), thus the differential genotype and treatment interactions between the male and female mice may be caused by sex hormone modulation of the KP.

### Locomotion and Motor Performance

Although motor deficits have not been the major area of focus in AD (unlike other dementias, such as Parkinson’s disease), they are one of the leading causes of death because AD patients often fall and break their bones or choke on their food because of the loss of the swallowing reflex (dysphagia). The 3xTg-AD mice show enhanced performance on the rotarod compared to the WT controls as early as 6 months of age (Stover et al., 2015a), and this continues into old age (Garvock-de Montbrun et al., 2019). Female mice also perform better than males; however, this is likely caused by the smaller body size of the female mice. We replicated these results in the present study because female mice performed better than males and female 3xTg-AD mice performed better than female WT mice. DWG-1036 did not have an effect on the performance of the 3xTg-AD mice. The *Tau* *_P301L_* mutation that occurs in 3xTg-AD mice has been shown to improve motor performance on the rotarod at early ages (Morgan et al., 2008). Hence, the motor improvement of the 3xTg-AD mice may be unrelated to Aβ pathology and increased KP activity, which explains the lack of DWG-1036 treatment effect on the 3xTg-AD mice. On the other hand, DWG-1036 improved the motor performance of the WT mice, which may be caused by the increased tryptophan levels caused by KP inhibition. This extra tryptophan can be converted into serotonin, and serotonergic antidepressant administration has been shown to improve performance of chronically stressed mice on the rotarod (Mizoguchi et al., 2002). Moreover, mice lacking the serotonin transporter (5-HTT) showed poor performance on the rotarod compared to the WT controls (Holmes et al., 2002).

### Anxiety and Depression

Increased irritability, anxiety, and depression-like symptoms are commonly observed in AD patients. These mood disturbances have a significant negative impact on the life of AD patients and their caregivers. In our study, changes in anxiety levels were measured by open arm entrance and freezing frequency in the EPM, and depression-like symptoms were measured by immobility in the tail suspension task. Previous studies using the EPM showed elevated anxiety levels in the 3xTg-AD mice (Sterniczuk et al., 2010; Pietropaolo et al., 2014; Zhang et al., 2016). In our study 3xTg-AD mice spent more time in the open arms of the EPM, which may be interpreted as reduced anxiety. However, it is important to consider any confounding variables, especially because the 3xTg-AD mice spent more than 50% of the time in the open arms, which indicates a preference rather than exploratory behavior. Jawhar et al. (2012) showed a similar trend in the 5xFAD mice, another commonly used mouse model of AD (Oakley et al., 2006), and interpreted the increased time in the open arms as reduced anxiety. Conversely, Flanigan et al. (2014) showed that interneuronal loss in the barrel field was causing painful whisker stimulation in the 5xFAD mice, which caused an avoidance for the closed arms in the EPM. To the best of our knowledge, the whisker barrel field of the 3xTg-AD mice has not been studied, but GABAergic neuronal loss has been shown in a mouse model carrying the same mutations (Loreth et al., 2012). In addition, we have measured differences in whisker movements, such as lower mean angular positions and retraction speeds in 3xTg-AD mice compared to WT controls (Simanaviciute et al., in press). Thus, the increased time spent in the open arms may be caused by painful whisker stimulation in the closed arms, and therefore, it is important to study other anxiety-related behaviors in the EPM. For freezing behavior, a similar trend to the results in trace fear conditioning was observed: although freezing frequency was decreased in the male 3xTg-AD mice treated with DWG-1036, there was no such effect of treatment for the females, which once again suggest a sexually dimorphic effect of DWG-1036, which improves the symptoms of males but not females.

The tail suspension test was used as a measure of learned helplessness and depression-like behavior, which is seen in both humans with AD (Chi et al., 2014) and mouse models (Nyarko et al., 2019), including the 3xTg-AD mice, and have been linked to impaired monoamine transmission (Romano et al., 2014). In this study, 3xTg-AD mice treated with DWG-1036 froze less frequently than the 3xTg-AD mice that received the vehicle, whereas there were no differences between the treatment groups in WT mice. As discussed above, inhibiting IDO/TDO may increase the serotonin levels, compensating for the decrease caused by other AD mechanisms. IDO and KP activity has been studied in depression independent of AD as well: although kynurenine and KA were decreased in patients with depression, IDO and quinolinic acid levels were elevated (Wichers and Maes, 2004; Ogyu et al., 2018). Hence, our results suggest that DWG-1036, and other agents decreasing KP activity, may be successful at reducing depression-like symptoms.

### DWG-1036 Side Effects

Although DWG-1036 treatment was successful at reversing or ameliorating some of the behavioral symptoms of AD, it also had some negative side effects. As shown by the tolerability study and throughout the treatment period, DWG-1036 caused weight loss, especially in the female mice, which may be a direct result of treatment or caused by other accompanying complications. Since IDO and TDO are the first and rate-limiting enzymes of the KP, DWG-1036 may cause excessive tryptophan accumulation or hypertryptophanemia (Ferreira et al., 2017). This may cause various disorders, such as excessive fatigue (Yamamoto et al., 2012), and if it occurs during development, intellectual disability, mood disorders, hypersexuality, and sensory deficits (Martin et al., 1995). Another possible outcome of decreasing KP activity is increased serotonin levels, which may cause serotonin syndrome-like symptoms in mice (Haberzettl et al., 2013). Moreover, serotonin is involved in feeding regulation (Magalhães et al., 2010), as serotonergic receptors 5-HT1B, 5-HT2C, and 5-HT6 mediate satiety (Voigt and Fink, 2015). This may also explain the weight loss observed in the mice treated with higher doses of DWG-1036. Moreover, the weight loss affected female mice more than male mice in this study. This may be caused by sex differences in body weight prior to treatment. As the female mice had lower body weights compared to the male mice, equal amounts of weight loss resulted in a higher percentage of the body weight loss for the females. In addition, in a preclinical population of individuals with high neocortical AB levels, higher serum KP metabolite levels compared to healthy controls were shown in women but not men (Chatterjee et al., 2018). This may explain the protection of female TG mice from toxicity because KP activity is elevated in TG females, and thus DWG-1036 may be normalizing their levels without causing hypertryptophanemia.

## Conclusions

After being treated with the novel IDO inhibitor DWG-1036 between 2 and 6 months of age, 3xTg-AD mice showed improvements in cognition as well as anxiety and depression-related behaviors. This shows the therapeutic potential of targeting the KP and tryptophan metabolism in AD. KP overactivity and metabolite-related neurotoxicity are downstream of increased Aβ, which potentially increases the window for therapeutic intervention: although treatments targeting KP would not decrease Aβ_42_ accumulation, they would decrease neurodegeneration. Thus, neurobiological studies on KP-related interventions should focus on neurodegeneration instead of Aβ42 clearance. Indeed, reduced neurodegeneration upon TDO inhibition has been shown in animal models of AD, Parkinson’s disease, and Huntington’s disease (Breda et al., 2016). Similarly, inhibition of kynurenine 3-monooxygenase has been shown to reduce synaptic loss in the APPtg mouse model of AD (Zwilling et al., 2011).

However, inhibiting IDO/TDO may not be the ideal method to target the KP. The neuroactive metabolites of the KP have opposing roles in neurodegenerative diseases: although 3-HK and QA contribute to neurotoxicity, KA is neuroprotective (Tan et al., 2012). Therefore, targeting individual metabolites may be more beneficial for developing treatments for AD. Both 3-HK and KA are synthesized from kynurenine with the enzymes kynurenine-3-monooxygenase (KMO) and kynurenine aminotransferase (KAT), respectively (Wang et al., 2012; **Figure 1**), which makes these enzymes valuable targets of intervention (Han et al., 2010; Smith et al., 2016). Moreover, 3-HK gets further metabolized into 3-hydroxyanthranilic acid, which gets metabolized to QA in microglia with a non-enzymatic reaction. Finally, QA is converted to NAD by quinolinate phosphoribosyl transferase (QPRT) in neurons and astrocytes. Hence, a combination of compounds inhibiting the activity of KMO and enhancing KAT and QPRT might have better outcomes in decreasing the neurotoxicity in AD and ameliorating the behavioral deficits. Even though tryptophan metabolism *via* the KP seems to be involved in the progression of AD, there are many other mechanisms underlying AD, including cholinergic (dys)function, metabolic deficits, and environmental factors (Grant et al., 2002; Dziewczapolski et al., 2009; Lee et al., 2018). In addition, thioredoxin-interacting protein (TXNIP) is increased by Aβ_42_ and increases oxidative stress, thereby increasing the progression of AD (Fertan et al., 2019a). Future studies should investigate the neurobiological mechanisms in which these factors contribute to AD and their interaction with each other and Aβ_42_.

## Data Availability

The datasets generated for this study are available on request to the corresponding author.

## Ethics Statement

The animal study was reviewed and approved by Dalhousie University Council of Animal Ethics.

## Author Contributions

EF: Conception and design, collection and assembly of data, analysis and interpretation, final approval of manuscript. AW and RB: Conception and design, analysis and interpretation, final approval of manuscript. MB, PS, BK, ED-C: Design and production of DWG-1036, preliminary pharmacodynamic testing of DWG-1036. KS and DW: Design and production of DWG-1036, preliminary pharmacodynamic testing of DWG-1036, conception and design.

## Funding

This work was supported by a Discovery grant from Natural Sciences and Engineering Research Council of Canada to REB (Grant RG7441) and an Ontario Neurodegenerative Diseases Research Initiative Basic Science Program grant form the Ontario Brain Institute to DFW.

## Conflict of Interest Statement

The authors declare that the research was conducted in the absence of any commercial or financial relationships that could be construed as a potential conflict of interest.
